# The Japan Public Health Center-based Prospective Study for the Next Generation (JPHC-NEXT): Study Design and Participants

**DOI:** 10.2188/jea.JE20180182

**Published:** 2020-01-05

**Authors:** Norie Sawada, Motoki Iwasaki, Taiki Yamaji, Atsushi Goto, Taichi Shimazu, Manami Inoue, Kozo Tanno, Kiyomi Sakata, Kazumasa Yamagishi, Hiroyasu Iso, Nobufumi Yasuda, Tadahiro Kato, Isao Saito, Maiko Hasegawa, Kiyoshi Aoyagi, Shoichiro Tsugane

**Affiliations:** 1 Epidemiology and Prevention Group, Center for Public Health Sciences, National Cancer Center, Tokyo, Japan; 2 Department of Hygiene and Preventive Medicine, Iwate Medical University, Iwate, Japan; 3 Department of Public Health Medicine, Faculty of Medicine, University of Tsukuba, Ibaraki, Japan; 4 Public Health, Department of Social Medicine, Osaka University Graduate School of Medicine, Osaka, Japan; 5 Department of Public Health, Kochi University Medical School, Kochi, Japan; 6 Center for Education and Educational Research, Faculty of Education, Ehime University, Matsuyama, Japan; 7 Department of Community Health Systems Nursing, Ehime University Graduate School of Medicine, Matsuyama, Japan; 8 Ken-nan Healthcare Office, Nagasaki, Japan; 9 Department of Public Health, Nagasaki University Graduate School of Biomedical Sciences, Nagasaki, Japan

**Keywords:** JPHC-NEXT, population-based cohort, genomic research

## Abstract

**Background:**

Lifestyle and life-environment factors have undergone drastic changes in Japan over the last few decades. Further, many molecular epidemiologic studies have reported that genetic, epigenetic, and other biomarker information may be useful in predicting individual disease risk.

**Methods:**

The Japan Public Health Center-based Prospective Study for the Next Generation (JPHC-NEXT) was launched in 2011 to identify risk factors for lifestyle-related disease, elucidate factors that extend healthy life expectancy, and contribute toward personalized healthcare based on our more than 20 years’ experience with the JPHC Study. From 2011 through 2016, a baseline survey was conducted at 16 municipalities in seven prefectures across the country. A self-administered questionnaire was distributed to all registered residents aged 40–74, which mainly asked about lifestyle factors, such as socio-demographic situation, personal medical history, smoking, alcohol and dietary habits. We obtained informed consent from each participant to participate in this long follow-up study of at least 20 years, including consent to the potential use of their residence registry, medical records, medical fee receipts, care insurance etc., and to the provision of biospecimens (blood and urine), including genomic analysis.

**Results:**

As of December 31, 2016, we have established a population-based cohort of 115,385 persons (Response rate 44.1%), among whom 55,278 (47.9% of participants) have provided blood and urine samples. The participation rate was slightly higher among females and in the older age group.

**Conclusion:**

We have established a large-scale population-based cohort for next-generation epidemiological study in Japan.

## INTRODUCTION

Elucidating preventive factors against barriers to a healthy life, such as cancer and cardiovascular disease—major causes of death which lower quality of life (QOL)—is important in expanding healthy life expectancy in Japan.^1^ Beginning in 1990, we have conducted the Japan Public Health Center-based Prospective Study (JPHC Study),^2^ consisting of 140,000 residents aged 40 through 69 years who lived within 11 public health center-based areas nationwide in 1990–1994. The JPHC Study has since yielded more than 300 papers published on associations between potential etiologic factors and the incidence of or mortality from cancer, cardiovascular disease and other diseases associated with shorter life expectancy.

However, lifestyle and life-environment factors among the Japanese population have undergone drastic changes during these decades,^3^ and many molecular epidemiological studies have reported associations between genetic and environmental factors and diseases^4^^–^^6^ and identified biomarkers that may be useful in predicting disease risk in individuals.^7^^,^^8^ The addition of such biomarker information to environmental factors will be helpful in establishing personalized healthcare.

Japan has experienced a rapid aging of the population, resulting from a decline in the birthrate.^1^ In 2017, the proportion of deaths due to cancer, heart disease, and cerebrovascular disease were 28.7%, 15.2%, and 9.4%, respectively. In addition, the 2016 Comprehensive Survey of Living Conditions in Japan showed that dementia and fractures or falls are the major causes of the need for assistance or long-term care, accounting for 24.8% and 10.8% of in-home recipients, respectively.^9^ Given that average life expectancy in Japan is already among the highest in the world^10^ and is increasing,^1^ efforts to extend healthy life expectancy will require the elucidation of preventive factors for not only life-style related diseases but also functional disability. Therefore, research on the extension of healthy life expectancy in an aging society is an urgent issue.

In 2011, we launched the JPHC Study for the Next Generation (JPHC-NEXT). This large-scale, population-based prospective study has been designed to identify risk factors for lifestyle-related disease, elucidate factors that extend healthy life expectancy, and contribute toward personalized healthcare.

## METHODS

### Organization

**Figure 1.**
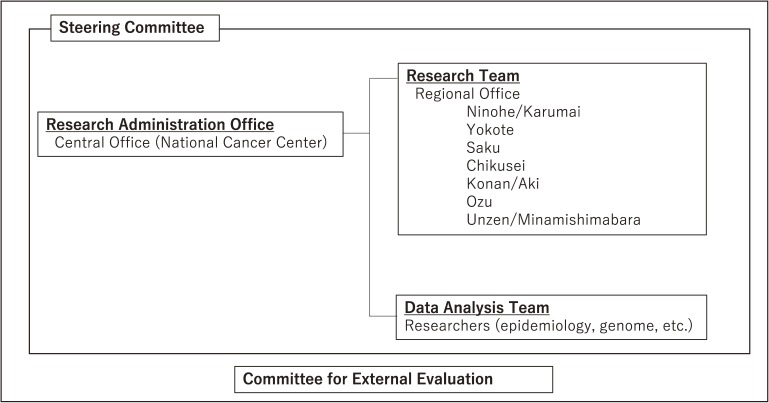
Organization of the Japan Public Health Center-based Prospective Study for the Next Generation (JPHC-NEXT)

The organization of JPHC-NEXT is listed in Figure 1. A Steering Committee was organized to manage and control the progress of the study. A central office was established at the National Cancer Center, and regional offices were established in each of the local areas involved. Regional offices were selected from among public health centers and universities able to play a role in managing collaboration with municipalities and hospitals in each area. The study protocol of JPHC-NEXT was developed by the research members of JPHC-NEXT and approved by the Institutional Review Board of the National Cancer Center, as well as at each collaborating university in the regional areas.

### Cohort subjects

**Figure 2.**
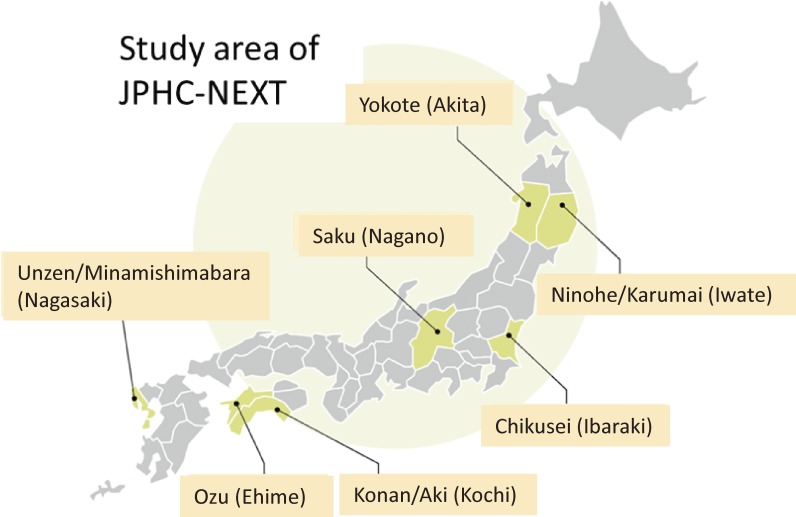
Map of the JPHC-NEXT Study

The JPHC-NEXT is being conducted under a population-based cohort design in 16 municipalities of seven prefectural areas across Japan, namely the Ninohe/Karumai area (Ninohe City and Karumai Town in Iwate Prefecture), Yokote area (Yokote City in Akita Prefecture), Saku area (Saku City, Sakuho Town, Koumi Town, Minamimaki Village, Minamiaiki Village, Kitaaiki Village and Kawakami Village in Nagano Prefecture), Chikusei area (Chikusei City in Ibaraki Prefecture), Konan/Aki area (Kagami and Noichi districts in Konan City and Aki City in Kochi Prefecture), Ozu area (Ozu City in Ehime Prefecture) and Unzen/Minamishimabara area (Unzen City and Minamishimabara City in Nagasaki Prefecture) (Figure 2). Among these seven areas, the Ninohe/Karumai, Yokote, Saku, and Konan areas are also participating in the JPHC Study. However, the areas of the Ninohe/Karumai, Yokote, and Saku municipalities are larger than they were in the JPHC Study because of municipal mergers occurring in 2005–2006. All areas were selected in consideration of geographical distribution, size, and feasibility.

The target population for the JPHC-NEXT is the total of 261,939 residents (130,602 men and 131,337 women) aged 40–74 years who were registered in the basic residence registers of the 16 municipalities at the time of enrollment in 2011–2016. The inclusion criteria for the JPHC-NEXT are persons who consent to participate in the study.

### Baseline survey

#### Questionnaire

We distributed a self-administered questionnaire to the entire target population at the time of enrollment and asked each participant to report their lifestyle and social factors, such as personal medical history, smoking, alcohol drinking, dietary habits, social support, social network, psychological health, educational level, and income.^11^ The dietary habits component of this questionnaire is a comprehensive validated food frequency questionnaire (FFQ) that includes 172 food items.^12^ We distributed the questionnaire via the following methods: 1) through related community organizations within each municipality, 2) at health check-ups, and 3) by mail. Incomplete answers were supplemented by telephone interview or mail; or, incomplete answers for participants of health check-ups were confirmed via face-to-face interview, telephone interview, or mail, in accordance with our manual.

Based on evidence from the JPHC Study, we encouraged responding participants to adopt healthy behaviors by providing each with individualized analyses of their personal data, titled “10-year cumulative probability of the occurrence of cancer and cardiovascular disease”^13^ and “Diet intake”. Additionally, we rewarded participants who answered the questionnaire with a prepaid shopping card of 1,000 yen equivalent value.

#### Blood and urine

We collected 7 mL of blood using an EDTA tube and 4 mL of urine from participants under informed consent on the following occasions. First, we collected biospecimens at specific health check-ups conducted for persons aged 40 through 74 years under the National Health Insurance. Second, to cover subjects who do not receive specific health check-ups, we also collected these biospecimens at comprehensive medical examinations or occasions organized specifically for blood and urine donation for this study. The tube was centrifuged for 10 min at 3,500–4,000 rpm as soon as possible after collection, and the plasma, erythrocyte, and buffy layers were divided into five 1.0 mL tubes (three for the plasma and one each for erythrocyte and buffy layers). Urine samples were stored in 4.0 mL tubes. All samples were stored at −80°C. Biospecimens were collected within about 1 year of the completion of the questionnaire.

Based on evidence from the JPHC Study,^14^ we provided subjects with the results for “10-year cumulative probability of gastric cancer occurrence,” obtained by measuring serum immunoglobulin G antibodies to *Helicobacter pylori* and blood levels of pepsinogen. Serums were derived from the biospecimens for the health examinations or the residuals of blood collected at occasions organized specifically for blood donation for this study. We stored the residuals of serum samples using the same method as for plasma.

### Follow-up system

Participants are being followed for vital status (or cause of death), migration, and the occurrence of cancer, other potentially lifestyle-related diseases, and need for support/long-term care certification.

#### Vital status and migration

Information on the vital status and migration of participants is centralized from municipalities in the research areas to the central office with the help of the regional offices. For participants who move out the research area, the central office refers to the certificate of residence and vital status at their new address with the consent of participants. The cause of death of participants is confirmed using death certificates in public health centers in each area, with the permission of the Ministry of Health, Labour and Welfare. Regional centers collect information on cause of death and send it to the central office annually.

#### Incidence of lifestyle-related diseases

The 2013 Cancer Registry Promotion Act made cancer reporting a legal requirement of hospitals from 2016. Therefore, a cancer registry is available to confirm the incidence of cancer among our study participants. However, the cancer registry does not include information on all cancer subtype variables that might influence risk, such as estrogen receptor status for breast cancer or Gleason score for prostate cancer and so on. Accordingly, incidence data of cancer from 2011 to 2015 and information of cancer subtypes were obtained using data from local major hospitals, in addition to population-based cancer registry data. Regarding cardiovascular diseases (coronary heart disease, stroke, congestive heart failure, and aortic disease), we first categorized candidate cases using the diagnosis from the inpatient medical record and/or medical expenses. A physician or researcher then extracted detailed information from the medical record, including imaging, into cohort-specific registration forms at major hospitals in the research areas. In addition, we defined subjects who needed Support/Long-Term Care certification and those with dementia using the public long-term care insurance, with disabling dementia identified in persons with disease of grade ≥IIa under this system, as previously reported.^15^ To confirm other lifestyle-related diseases, we are currently considering the use of electronic medical records and medical expenses.

### Follow-up surveys

**Figure 3.**
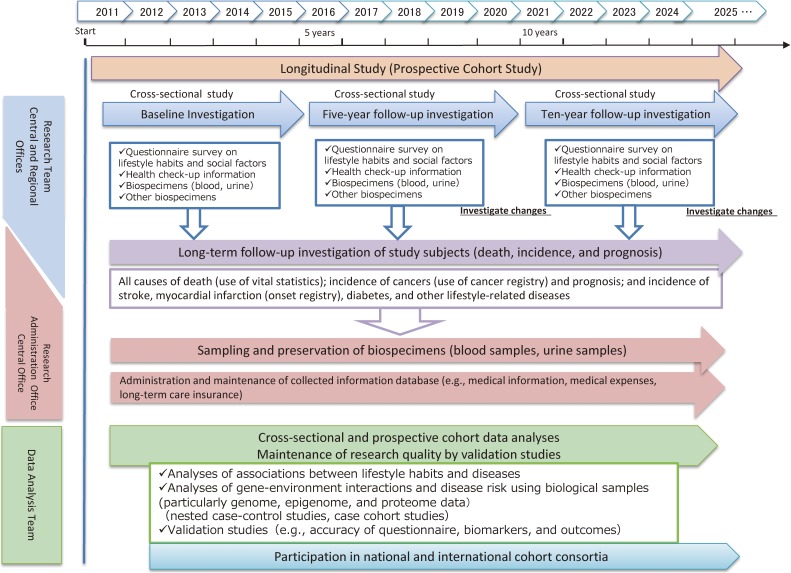
Schedule of the JPHC-NEXT Study

After the baseline survey, two follow-up surveys are scheduled to be conducted at 5-year intervals to assess changes in lifestyle and life-environmental factors, including diet and disease history, using a questionnaire. To objectively evaluate changes in lifestyle, health, DNA methylation, and other factors, we are planning to collect blood and urine every 5 years. The research schedule is shown in Figure 3.

### Informed consent

Signed informed consent was obtained from all participants to participate in this long follow-up study, including consent for the collection of information on medical history, medical expenses, care insurance, support/long-term care certification, cancer registry, residence registry, and death certificate, and for the use of biospecimens for research, including genome analysis.

## RESULTS

### Participants

**Figure 4.**
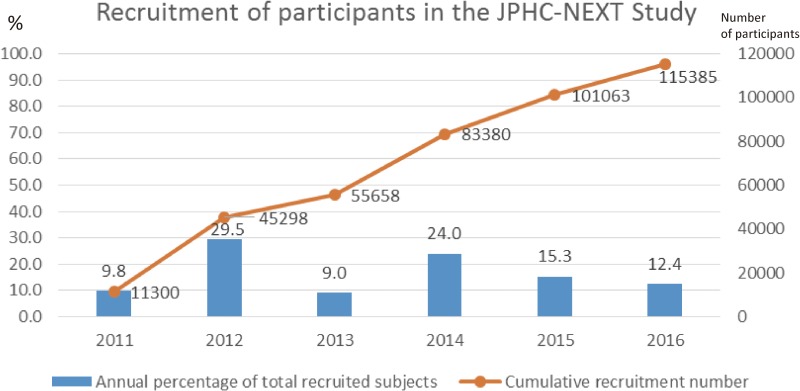
Recruitment of participants in the JPHC-NEXT Study

Among a total of 261,939 residents (130,602 men and 131,337 women) aged 40–74 years in the research areas, 115,385 persons (44.1% of total residents; 53,210 men and 62,175 women) consented to participate in the study. The consent rate in women (47.3%) was higher than that in men (40.7%) (Table 1, Table 2, and Table 3). The area-specific proportion of consent ranged from 14.7% to 74.5% in total participants, from 14.2% to 71.4% in men, and from 15.2% to 81.7% in women. The median recruitment year was 2014 (Figure 4).

**Table 1.** Population in municipalities and number of participants in JPHC-NEXT

Regional Office (Recruitment Period)	Study Area	Public Health Center (PHC)		Total population in Study Area (a)	Number of participants (b)	Proportion of participants (b/a*100) (%)	Method of questionnaire distribution ^*^1	Number of subjects who provided biospecimens (c)	Proportion of subjects who provided biospecimens (c/b*100) (%)	Method of biospecimen collection ^*^2
Iwate Medical University	Ninohe/Karumai Area in Iwate Prefecture		Subtotal	18,747	10,572	56.4		4,228	40.0	
(2015–2016)	Ninohe City	Ninohe PHC		13,895	7,434	53.5	1)	2,887	38.8	1) and 3)
	Karumai Town			4,852	3,138	64.7	1)	1,341	42.7	1) and 3)

Yokote Public Health Center	Yokote Area in Akita Prefecture		Subtotal	45,964	29,876	65.0		16,016	53.6	
(2011–2014)	Yokote City	Yokote PHC		45,964	29,876	65.0	1) and 2), partly 3)	16,016	53.6	1), 2) and 3)

Saku Public Health Center	Saku Area in Nagano Prefecture		Subtotal	56,439	31,478	55.8		13,068	41.5	
(2011–2012)	Saku City	Saku PHC		44,344	23,413	52.8	1), partly 3)	8,662	37.0	1), 2) and 3)
	Sakuho Town			5,620	3,693	65.7	1), partly 3)	2,103	56.9	1), 2) and 3)
	Koumi Town			2,385	1,747	73.2	1), partly 3)	703	40.2	1), 2) and 3)
	Minamimaki Village			1,434	717	50.0	1), partly 3)	470	65.6	1), 2) and 3)
	Kitaaiki Village			345	257	74.5	1), partly 3)	143	55.6	1), 2) and 3)
	Minamiaiki Village			474	353	74.5	1), partly 3)	210	59.5	1), 2) and 3)
	Kawakami Village			1,837	1,298	70.7	1), partly 3)	777	59.9	1), 2) and 3)

Osaka University and University of Tsukuba	Chikusei Area in Ibaraki Prefecture		Subtotal	55,003	17,323	31.5		10,101	58.3	
(2011–2016)	Chikusei City	Chikusei PHC		55,003	17,323	31.5	2) and 3), partly 1)	10,101	58.3	1)

Kochi University	Konan/Aki Area in Kochi Prefecture		Subtotal	20,434	7,455	36.5		3,777	50.7	
(2011–2014)	Kagami and Noichi districts in Konan City	Chuohigashi PHC		11,151	3,708	33.3	1), partly 3)	1,567	42.3	1) and 3)
	Aki City	Aki PHC		9,283	3,747	40.4	1) and 2), partly 3)	2,210	59.0	1) and 3)

Ehime University	Ozu Area in Ehime Prefecture		Subtotal	21,421	6,999	32.7		3,536	50.5	
(2014–2016)	Ozu City	Yahatahama PHC		21,421	6,999	32.7	2) and 3), partly 1)	3,536	50.5	1)

Nagasaki University	Unzen/Minamishimabara Area in Nagasaki Prefecture		Subtotal	43,931	11,682	26.6		4,552	39.0	
(2014–2016)	Unzen City	Kennan PHC		21,135	3,107	14.7	3)	1,686	54.3	1) and 3)
	Minamishimabara City			22,796	8,575	37.6	1), partly 3)	2,866	33.4	1) and 3)

			**Total**	**261,939**	**115,385**	**44.1**		**55,278**	**47.9**	

^*^1: 1) through related community organizations within each municipality 2) at a health check-ups 3) by mail.

^*^2: 1) health check-ups, 2) comprehensive medical examination 3) storage center for biospecimens collected for this study.

**Table 2.** Population in municipalities and number of participants in JPHC-NEXT, males

Regional Office	Study Area		Total population in Study Area (a)	Number of participants (b)	Proportion of participants (b/a*100) (%)	Number of subjects who provided biospecimens (c)	Proportion of subjects who provided biospecimens (c/b*100) (%)
Iwate Medical University	Ninohe/Karumai Area in Iwate Prefecture	Subtotal	9,498	4,954	52.2	1,750	35.3
	Ninohe City		6,941	3,434	49.5	1,169	34.0
	Karumai Town		2,557	1,520	59.4	581	38.2

Yokote Public Health Center	Yokote Area in Akita Prefecture	Subtotal	22,574	14,011	62.1	7,138	50.9
	Yokote City		22,574	14,011	62.1	7,138	50.9

Saku Public Health Center	Saku Area in Nagano Prefecture	Subtotal	28,581	14,881	52.1	5,807	39.0
	Saku City		22,410	11,034	49.2	3,826	34.7
	Sakuho Town		2,851	1,776	62.3	960	54.1
	Koumi Town		1,195	813	68.0	309	38.0
	Minamimaki Village		743	328	44.1	198	60.4
	Kitaaiki Village		175	125	71.4	60	48.0
	Minamiaiki Village		245	166	67.8	91	54.8
	Kawakami Village		962	639	66.4	363	56.8

Osaka University and University of Tsukuba	Chikusei Area in Ibaraki Prefecture	Subtotal	27,856	7,812	28.0	4,102	52.5
	Chikusei City		27,856	7,812	28.0	4,102	52.5

Kochi University	Konan/Aki Area in Kochi Prefecture	Subtotal	9,942	3,308	33.3	1,561	47.2
	Kagami and Noichi districts in Konan City		5,395	1,616	30.0	646	40.0
	Aki City		4,547	1,692	37.2	915	54.1

Ehime University	Ozu Area in Ehime Prefecture	Subtotal	10,552	2,955	28.0	1,324	44.8
	Ozu City		10,552	2,955	28.0	1,324	44.8

Nagasaki University	Unzen/Minamishimabara Area in Nagasaki Prefecture	Subtotal	21,599	5,289	24.5	1,914	36.2
	Unzen City		10,463	1,482	14.2	782	52.8
	Minamishimabara City		11,136	3,807	34.2	1,132	29.7

		**Total**	**130,602**	**53,210**	**40.7**	**23,596**	**44.3**

**Table 3.** Population in municipalities and number of participants in JPHC-NEXT, females

Regional Office	Study Area		Total population in Study Area (a)	Number of participants (b)	Proportion of participants (b/a*100) (%)	Number of subjects who provided biospecimens (c)	Proportion of subjects who provided biospecimens (c/b*100) (%)
Iwate Medical University	Ninohe/Karumai Area in Iwate Prefecture	Subtotal	9,249	5,618	60.7	2,478	44.1
	Ninohe City		6,954	4,000	57.5	1,718	43.0
	Karumai Town		2,295	1,618	70.5	760	47.0

Yokote Public Health Center	Yokote Area in Akita Prefecture	Subtotal	23,390	15,865	67.8	8,878	56.0
	Yokote City		23,390	15,865	67.8	8,878	56.0

Saku Public Health Center	Saku Area in Nagano Prefecture	Subtotal	27,858	16,597	59.6	7,261	43.7
	Saku City		21,934	12,379	56.4	4,836	39.1
	Sakuho Town		2,769	1,917	69.2	1,143	59.6
	Koumi Town		1,190	934	78.5	394	42.2
	Minamimaki Village		691	389	56.3	272	69.9
	Kitaaiki Village		170	132	77.6	83	62.9
	Minamiaiki Village		229	187	81.7	119	63.6
	Kawakami Village		875	659	75.3	414	62.8

Osaka University and University of Tsukuba	Chikusei Area in Ibaraki Prefecture	Subtotal	27,147	9,511	35.0	5,999	63.1
	Chikusei City		27,147	9,511	35.0	5,999	63.1

Kochi University	Konan/Aki Area in Kochi Prefecture	Subtotal	10,492	4,147	39.5	2,216	53.4
	Kagami and Noichi districts in Konan City		5,756	2,092	36.3	921	44.0
	Aki City		4,736	2,055	43.4	1,295	63.0

Ehime University	Ozu Area in Ehime Prefecture	Subtotal	10,869	4,044	37.2	2,212	54.7
	Ozu City		10,869	4,044	37.2	2,212	54.7

Nagasaki University	Unzen/Minamishimabara Area in Nagasaki Prefecture	Subtotal	22,332	6,393	28.6	2,638	41.3
	Unzen City		10,672	1,625	15.2	904	55.6
	Minamishimabara City		11,660	4,768	40.9	1,734	36.4

		**Total**	**131,337**	**62,175**	**47.3**	**31,682**	**51.0**

Among participants, 55,278 persons consented to the use of their biospecimens for this research, including genome analysis (47.9%). The proportion of persons who provided biospecimens was higher in women (51.0%) than in men (44.3%). The range of area-specific proportion of provision of biospecimens among participants was from 33.4% to 65.6% in total participants, from 29.7% to 60.4% in men, and from 36.4% to 69.9% in women. The proportion of specimens provided by women (31,682 women, 57.3%) was higher than that by men (23,596 men, 42.7%).

**Figure 5.**
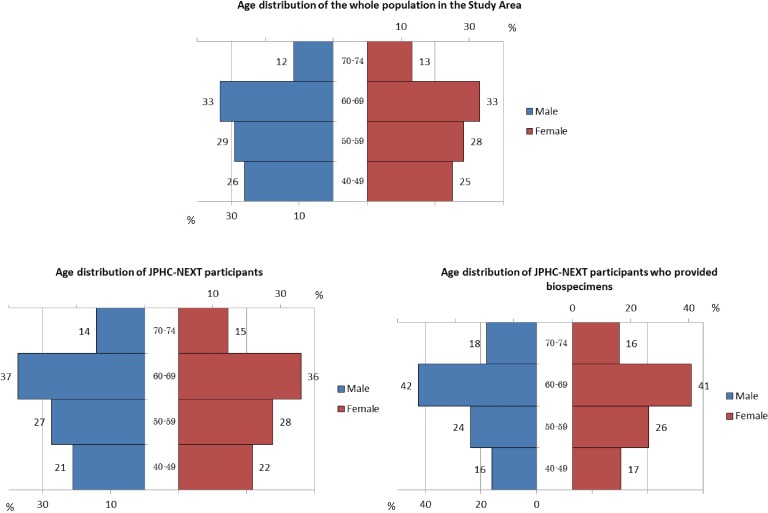
Age distribution of the study area and participants in the JPHC-NEXT Study

Regarding age distribution, participants in the JPHC-NEXT were slightly older than the overall population in the study area, and a slightly higher proportion of older subjects provided biospecimens (Figure 5).

We compared the baseline characteristics of JPHC-NEXT and JPHC subjects in the same age group (40–59 years). Among these subjects, men in JPHC-NEXT tended to smoke less and drink less alcohol than those in the JPHC Study, while women tended to smoke more and drink more alcohol. Mean height and weight were higher in JPHC-NEXT than in the JPHC Study in both men and women. Although mean BMI in men was higher in JPHC-NEXT than in the JPHC Study, mean BMI in women was lower. Mean age at menarche was lower in JPHC-NEXT than in the JPHC Study (eTable 1).

## DISCUSSION

The JPHC-NEXT incorporates 16 study areas (city, town, or village level) from 7 prefectures. The participation rate has tended to be higher in towns and villages than in the cities, and varied with the method of questionnaire distribution. When questionnaires were distributed by local community organizations administered by or associated with the respective municipality, the participation rate was moderately high, but when questionnaires were distributed by mail, it was very low (less than 20%). We tried to distribute the questionnaire by hand, but some municipalities had only small communities and we could not access all target areas by hand. Even though local community organizations distributed questionnaire at the time of JPHC Study in the same areas, the members of active organizations had substantially decreased over the subsequent 20 years. As in the JPHC Study, participation rates were lower in men than in women.^16^

The specific health check-ups conducted at annual community health examinations, which are conducted by municipalities,^17^ represent an efficient means of sourcing biospecimens and to obtain informed consent in a face-to-face manner, because these involve the assembly at a specific date and place of large numbers of people who are registered to receive National Health Insurance. However, collection at these health check-ups might lead to a degree of bias, because they are more frequently attended by women than men, who more commonly undergo health check-ups at their workplace or at a comprehensive medical examination supported by their employer. Indeed, we saw a gender difference in the collection rates of blood samples (62.9% in women and 37.1% in men) in the JPHC.^16^ To attenuate such bias in the JPHC-NEXT, we also collected biospecimens at comprehensive medical examinations or organized occasions specifically for blood and urine donation for this study. Bias was accordingly less than with the JPHC, albeit that the proportion of participants providing biosamples was still higher in women (57.3% in women, 42.7% in men). Although our participation rate was not particularly high (44.1% of the total target population), the age distribution of participants of the JPHC-NEXT was similar to that in the overall population in the study areas. Accordingly, the JPHC-NEXT is not biased with regard to age distribution. However, we cannot rule out the possibility that the moderate participation rate might have lead to a degree of bias with regard to lifestyle, incidence, or mortality. Additionally, subjects who provided biospecimens were slightly biased toward older age. Moreover, people who attend health check-ups tend to be health-conscious. Any generalization of the results should, therefore, be done with caution.^18^^,^^19^

Response rate in the JPHC Study was higher (81%)^2^ than that in JPHC-NEXT. In the 1990s, no ethical guidelines for epidemiological studies were available in Japan. Collection of written informed consent was not necessary; rather, response to a questionnaire was recognized as consent. This represents a substantial difference between JPHC and JPHC-NEXT. The lower response rate in JPHC-NEXT partly suggests that the provision of informed consent was a burden for participants.

The JPHC-NEXT uses the same or a similar questionnaire to those of the Tohoku Medical Megabank Project^20^ and some other cohort studies, such as the Murakami Cohort Study,^21^ Uonuma cohort,^22^ Yamagata study,^23^ and Chiba cohort.^24^ Additionally, we have considered how to integrate our questionnaire with the slightly different questionnaire used by the Japan Multi-Institutional Collaborative Cohort Study (J-MICC Study).^25^ Collaboration and integration with domestic and international genome cohorts is important in achieving our aim of contributing to the development of personalized healthcare.

In conclusion, the JPHC-NEXT has been undertaken to reveal risk factors for lifestyle-related disease and to contribute toward personalized healthcare. The JPHC-NEXT Study is expected to provide evidence for the extension of healthy life expectancy of future generations of the Japanese population.

## APPENDIX A. SUPPLEMENTARY DATA

eTable 1. Baseline characteristics in subjects aged 40–59 in JPHC and JPHC-NEXT
