# Supplementary material for: The Japan Public Health Center-based Prospective Study for the Next Generation (JPHC-NEXT): Study Design and Participants
Source: J Epidemiol. 2020 Jan 5;30(1):46–54. doi: 10.2188/jea.JE20180182 (PMC6908844; doi:10.2188/jea.JE20180182)
Supplement: Supplementary file 1 [file je-30-046-s001.pdf]

eTable 1. Baseline characteristics in subjects aged 40−59 in JPHC and JPHC-NEXT

	Male		Female	
	JPHC-NEXT	JPHC	JPHC-NEXT	JPHC
Year of birth, median (min-max)	1963 (1951−1977)	1942 (1930–1954)	1963 (1951–1977)	1942 (1930–1954)
Current smoker, %	42.6	54.5	11.5	8.3
Regular drinker, 1−2 times/week, %	66.0	70.3	30.7	16.0
Height, mean (SD)	170.2 (6.1)	164.8 (6.2)	157.0 (5.8)	152.6 (5.5)
Weight, mean (SD)	69.9 (12.1)	64.1 (9.0)	55.7 (10.5)	54.3 (7.8)
Body mass index, mean (SD)	24.2 (21.2)	23.6 (3.0)	23.0 (30.0)	23.3 (3.3)
Age at menarche, mean (SD)	-	-	12.8 (2.2)	14.3 (1.8)

JPHC, Japan Public Health Center-based Prospective Study; JPHC-NEXT, Japan Public Health Center-based Prospective Study for the Next Generation; SD, standard deviation.
